# The association of serum uric acid/albumin ratio with the development of coronary collateral circulation in patients with chronic total occluded coronary arteries

**DOI:** 10.34172/jcvtr.2023.31627

**Published:** 2023-03-16

**Authors:** Faysal Şaylık, Tufan Çınar, Remzi Sarıkaya, Tayyar Akbulut, Murat Selçuk, Emrah Özbek, Halil İbrahim Tanboğa

**Affiliations:** ^1^Department of Cardiology, Van Education and Research Hospital, Van, Turkey; ^2^Department of Cardiology, Sultan Abdulhamid Han Education and Research Hospital, Istanbul, Turkey; ^3^Department of Cardiology and Biostatistics, Istanbul Nisantasi University, Istanbul, Turkey

**Keywords:** Rentrop Score, Chronic Total Occlusion, Coronary Collateral Circulation, Uric Acid/ Albumin Ratio

## Abstract

***Introduction:*** Coronary collateral circulation (CCC) develops in chronic total occluded (CTO) vessels and protects the myocardium against ischemia in addition to the improvement of cardiac functions. Poor CCC is related to adverse cardiac events as well as poor prognosis. Serum uric acid/albumin ratio (UAR) has emerged as a novel marker associated with poor cardiovascular outcomes. We aimed to investigate whether there was an association between UAR and poor CCC in CTO patients.

***Methods:*** This study was comprised of 212 patients with CTO (92 with poor CCC and 120 with good CCC). All patients were graded based on Rentrop scores to poor CCC (Rentrop scores 0 and 1) and good CCC (Rentrop scores 2 and 3).

***Results:*** Poor CCC patients had higher frequencies of diabetes mellitus, triglyceride levels, Syntax and Gensini scores, uric acid, and UAR and lower lymphocyte, high-density lipoprotein cholesterol, and ejection fraction when compared to good CCC patients. UAR was an independent predictor of poor CCC in CTO patients. Furthermore, UAR had a better discriminative ability for patients with poor CCC from good CCC compared to serum uric acid and albumin.

***Conclusion:*** Based on the results of the study, the UAR could be used to detect poor CCC in CTO patients.

## Introduction

 Coronary chronic total occlusion (CTO) is defined by a high burden of atherosclerotic plaque, leading to complete obstruction of the coronary vessel for at least three months.^1^ Coronary collateral circulation (CCC), which develops as a protective mechanism against critical occlusion, could play a significant role in decreasing ischemic area and improving cardiac functions.^2^ The underlying possible factors for the formation of CCC have not been fully understood. However, inflammation, ischemia, growth factors, intravascular pressure, and genetic factors are thought to be responsible for the heterogeneity of CCC development in CTO patients.^2-5^

 The final product of purine metabolism, serum uric acid (UA), has a significant role in the occurrence and severity of coronary artery disease (CAD).^6^ It has also been reported that serum UA was higher in CTO patients with poor CCC.^2^ On the other hand, albumin is a well-known negative acute phase reactant, and its lower levels were found to be related to several cardiovascular diseases.^7^ Ischemic-modified albumin level were reported to be lower in poor CCC and was an independent predictor of poor CCC in previous reports.^8,9^ Serum uric acid/albumin ratio (UAR) has emerged as a novel marker that was associated with cardiovascular events in recent reports.^10,11^ The role of UAR in the CCC of CTO patients has not been evaluated previously in the literature. Thus, we aimed to investigate the association of serum UAR with CCC in CTO patients in this study.

## Materials and Methods

###  Patient selection

 This retrospective cross-sectional study consisted of 212 patients who were diagnosed with stable CAD between February 2014 and December 2021 and had a total occlusion of at least one major coronary artery on coronary angiogram based on the recommendation of the European Society of Cardiology. Patients with malignancy, chronic renal or liver disease, active infection, chronic inflammatory or autoimmune disease, using drugs that could affect serum UA levels, and gout disease were excluded from the study. The study was accepted by the local ethics committee and carried out in line with the 2008 revision of the Declaration of Helsinki.

###  Definitions 

 Diabetes mellitus (DM) was identified as postprandial glucose > 200 mg/dL, fasting blood glucose > 126 mg/dL, or the use of anti-diabetic medication. Systolic blood pressure of at least 140 mm Hg, diastolic blood pressure of at least 90 mm Hg, or use of anti-hypertensive medications were considered to be evidence of hypertension. Hyperlipidemia was accepted as having total cholesterol levels above 200 mg/dL or using anti-hyperlipidemic agents. Smokers were defined as patients who had smoked for at least 6 months during the previous year. Heart failure was accepted as having a left ventricular ejection fraction (LVEF) < 50 % with signs and symptoms of heart failure. Body mass index (BMI) was calculated by dividing weight (kg) by height(m)^2^.

###  Angiographic procedure

 Coronary angiography (CAG) was performed using the Judkins technique via radial or femoral access according to the cardiologist’s preference. Two experienced interventional cardiologists evaluated all CAG images. CTO was described as total blockage of antegrade flow or limited contrast transmission through the occluded lesion on the CAG. Syntax score (SS) was used to detect the extent of coronary artery stenosis and was calculated using a web calculator by two cardiologists.^12^ The intra- and inter-observer variabilities for SS were 2% and 3%, respectively. Gensini score was calculated for all patients as previously determined.^13^ Rentrop classification was used to assess the CCC grades as follows; grade 0 = no visible filling of the coronary collateral artery, grade 1 = filling of the collateral coronary arteries without reaching the epicardial regions, grade 2 = filling of the collateral arteries with partial reaching the epicardial regions, grade 3 = complete filling.^14^ The highest Rentrop grade was accepted when there were multiple CCCs. Finally, the study population was divided into two groups based on Rentrop classification as poor CCC (grades 0 and 1) and good CCC (grades 3 and 4). The intra- and inter-observer variabilities for Rentrop classification were 1% and 3%, respectively.

###  Electrocardiography

 Using an electrocardiography (ECG) machine, all ECGs were recorded in the resting condition at 512 Hz frequency and 25 mm per second paper speed (Nihon Kohden 1250). The QT interval is defined as the time between the start of the QRS and the end of the T wave, which occurs when the tangent to the T wave downslope intersects the isoelectric line. The period of time between the T wave’s peak and its end is referred to as the Tp-e interval.

###  Echocardiography

 All participants underwent echocardiographic assessment using a Vivid 7 GE (GE Healthcare, Little Chalfont, UK) echocardiography equipment from the left lateral decubitus position, as recommended by the American Society of Echocardiography.^15^ The modified Simpson method was used to compute the left ventricular ejection fraction (LVEF). M-mode echocardiography was used to investigate the interventricular septum diameter (IVS), LV end-systolic diameter (LVESD), and LV end-diastolic diameter (LVEDD). Conventional Doppler parameters such as late diastolic A wave and early diastolic E wave were traced by putting a sample volume at the mitral valve level. Tissue Doppler measurements, including early diastolic e waves and late diastolic a waves from left ventricular septal and lateral walls, were obtained by inserting sample volume at the junction of the mitral annulus of the left ventricle. The interval between the midpoint of aortic closure and the onset of e velocity was termed as the isovolumetric relaxation time (IVRT). The time interval between the peak of the E-wave and its predicted point on the baseline is defined as deceleration time (DT).

###  Laboratory analysis

 Upon admission to the cardiology clinic, blood samples were gained from an antecubital vein. A Beckman Coulter LH 780 hematology analyzer (Beckman Coulter, Miami, FL, USA) was used to assess hematologic parameters, and a Roche Cobas 6000 c501 (Roche, Mannheim, Germany) was used to analyze biochemical parameters. UAR was calculated by dividing serum UA by albumin. In our institution, the normal range of serum UA is 1.5 - 7.06 mg/dL, and albumin is 3.5 - 5.5 g/dL.

###  Statistical analysis

 An R program version 3.6.3 was used to calculate all statistical analyses (R statistical software, Institute for Statistics and Mathematics, Vienna, Austria). In order to determine if the variables were normally distributed, the Kolmogorov-Smirnov test was utilized. The continuous variables with normally distributed were denoted with a mean (SD), and without normally distributed with median (Q1-Q3). Numbers and percentages were utilized for categoric variables. For the comparison of continuous variables between the groups, the independent Student’s t-test and Mann-Whitney U tests were computed. Depending on the case, either the χ^2^ test or Fisher’s exact test was used to compare the categorical variables between the groups. Univariable logistic regression analysis was utilized to evaluate the relationship. between variables and poor CCC. Clinically significant factors that had a p-value of 0.05 or lower in the univariable logistic regression analysis were used in the multivariable logistic regression analysis. In regression models, Firth’s penalization likelihood method was employed to reduce overestimation. The model did not contain variables that had multicollinearity which was discovered by the logistic regression analysis (variance inflation factor > 3 or tolerance < 0.1). The multivariable model did not include UAR together with serum UA and albumin to prevent multicollinearity and interaction. Receiver operating curves (ROC) were used to compare the discrimination abilities of serum UA, albumin, and UAR for patients with poor CCC from good CCC using the De-Long test. The Box-plot graphic was employed to demonstrate the UAR levels between the Rentrop score grades. The 95 % confidence interval (CI) was used to examine the results, and a 2-tailed p-value of 0.05 was accepted as the significant level.

## Results

 Of 212 patients, 120 patients had good CCC (median age = 62 [49-68], 65.8% male) and 92 patients had poor CCC (median age = 60[52.5-68], 75 % male). The poor CCC group had higher rates of heart failure, higher triglycerides, higher serum UA, lower lymphocyte, and lower high-density cholesterol (HDL) compared to the good CCC group. The UAR was significantly higher in the poor CCC group than in the good CCC group. The Syntax and Gensini scores were both detected as higher in the poor CCC group when compared to the good CCC group. Other demographic and laboratory features of patients were presented in Table 1.

**Table 1:** Baseline comparison of demographic and laboratory findings between study groups

	**Good CCC (n=120)**	**Poor CCC (n=92)**	***P*** ** value**
Age, years	62.0 (49.0-68.0)	60.0 (52.5-68.0)	0.601
Male gender, n (%)	79 (65.8)	69 (75.0)	0.197
BMI, kg/m2	27.5 (25.0-29.4)	27.8 (25.9-29.7)	0.283
Risk factors			
Cigarette smoking, n (%)	62 (51.7)	48 (52.2)	1.000
Diabetes mellitus, n (%)	29 (24.2)	37 (40.2)	0.019
Hypertension, n (%)	54 (45.0)	40 (43.5)	0.935
Previous CAD, n (%)	33 (27.5)	33 (35.9)	0.248
Hyperlipidemia, n (%)	30 (25.0)	28 (30.4)	0.469
CHF, n (%)	46 (38.3)	52 (56.5)	0.013
Laboratory data			
WBC, x103/ µl	7.97 (6.96-9.87)	8.34 (7.07-9.69)	0.652
Hemoglobin, g/dL	14.9 (13.7-16.1)	14.7 (13.8-15.6)	0.239
Platelets, x103/ µl	244 (217-288)	226 (180-291)	0.117
RDW, %	42.8 (40.6-46.1)	44.6 (41.8-48.0)	0.060
Lymphocyte, x103/ µl	3.35 (2.96-4.22)	1.79 (1.53-3.77)	< 0.001
Monocyte, x103/ µl	0.53 (0.44-0.68)	0.49 (0.43-0.70)	0.352
MPV, fL	10.2 (9.28-10.5)	10.1 (8.70-10.8)	0.934
Creatinine, mg/dL	0.96 (0.76-1.08)	0.88 (0.79-0.99)	0.288
Calcium, mg/dL	9.27 (8.65-9.70)	9.13 (8.63-9.53)	0.222
Triglyceride, mg/dL	142 (83.3-194)	182 (149-276)	< 0.001
HDL cholesterol, mg/dL	42.0 (33.2-49.2)	38.2 (29.5-44.0)	0.014
LDL cholesterol, mg/dL	109 (91.8-142)	112 (91.1-130)	0.374
Uric acid, mg/dL	4.22 (1.35)	6.58 (1.76)	< 0.001
Albumin, g/dL	4.20 (3.86-4.60)	4.04 (3.80-4.50)	0.132
UAR	1.03 (0.85-1.19)	1.62 (1.30-1.95)	< 0.001
Drugs, n (%)			
Anti-platelets	54 (45.0)	53 (57.6)	0.093
ACE inh/ARBs	51 (42.5)	41 (44.6)	0.872
Beta blockers	70 (58.3)	57 (62.0)	0.695
Statins	38 (31.7)	34 (37.0)	0.509

Abbreviations; CCC: coronary collateral circulation, BMI: body mass index, CAD: coronary artery disease, CHF: congestive heart failure, WBC: white blood cell, RDW: red cell distribution width, MPV: mean platelet volume, HDL: high-density lipoprotein, LDL: low-density lipoprotein, UAR: serum uric acid/albumin ratio, ACE inh: angiotensin-converting enzyme inhibitors, ARBs: angiotensin-receptor blockers. *P* < 0.05 statistically significant.

Table 2 demonstrated the comparisons of ECG and echocardiographic parameters between groups. Heart rate, LVEF, and TDI lateral and septal e waves were lower in the poor CCC group, whereas LVEDD, LVESD, and IVRT were higher in the poor CCC group than in the good CCC group. The number of vessels with coronary stenosis was higher in the poor CCC group compared to the good CCC group. The poor CCC patients had a higher frequency of CTO in the left anterior descending artery and circumflex artery and a lower frequency of CTO in right coronary artery than patients with good CCC.

**Table 2:** Comparison of electrocardiographic and echocardiographic features between study groups

	**Good CCC (n=120)**	**Poor CCC (n=92)**	***P*** ** value**
Electrocardiographic data			
Heart rate, beat/min	79.0 (74.0-85.0)	76.0 (73.5-81.0)	0.015
QRS, msn	87.5 (83.0-95.0)	88.0 (80.0-92.0)	0.362
QTc, msn	408 (398-413)	404 (390-413)	0.149
Tpe, msn	75.0 (70.0-80.0)	75.0 (70.0-85.0)	0.668
Echocardiographic data			
LVEF, %	50.0 (45.0-55.0)	40.0 (40.0-45.0)	< 0.001
IVS, mm	13.0 (12.0-14.0)	13.0 (12.0-14.0)	0.175
LVEDD, mm	52.0 (50.0-55.2)	55.0 (50.0-61.0)	0.002
LVESD, mm	29.0 (28.0-35.0)	35.0 (28.0-40.0)	< 0.001
Mitral E, cm/s	0.72 (0.47)	0.69 (0.21)	0.482
Mitral A, cm/s	0.84 (0.14)	0.83 (0.15)	0.758
TDI lateral e, cm/s	9.00 (8.00-11.0)	9.00 (5.75-10.0)	0.007
TDI septal e, cm/s	8.00 (7.00-9.00)	7.00 (5.00-9.25)	0.014
TDI lateral a, cm/s	11.0 (9.00-13.0)	12.0 (9.00-15.0)	0.535
TDI septal a, cm/s	10.0 (7.00-11.0)	10.0 (8.00-11.0)	0.378
IVRT, ms	100 (92.0-109)	106 (94.0-121)	0.024
DT, ms	186 (155-196)	190 (154-204)	0.399
Angiographic findings			
Number of diseased vessels, n (%)	2.00 (2.00-3.00)	3.00 (2.00-4.00)	0.001
Syntax Score	14.0 (12.0-21.0)	21.0 (16.0-30.0)	< 0.001
Gensini score	50.0 (38.8-64.0)	60.5 (50.0-76.0)	< 0.001
CTO vessel, n (%)			
LAD	30 (25.0)	31 (33.7)	
Cx	44 (36.7)	44 (47.8)	0.007
RCA	46 (38.3)	17 (18.5)	

Abbreviations; CCC: coronary collateral circulation, LVEF: left ventricular ejection fraction, IVS: interventricular septum, LVEDD: left-ventricular end-diastolic diameter, LVESD: left-ventricular end-systolic diameter, TDI: tissue Doppler imaging, IVRT: isovolumetric relaxation time, DT: deceleration time, CTO: chronic total occlusion, LAD: left-anterior descending, Cx: circumflex, RCA: right coronary artery. *P* < 0.05 statistically significant.

**Figure 1:**
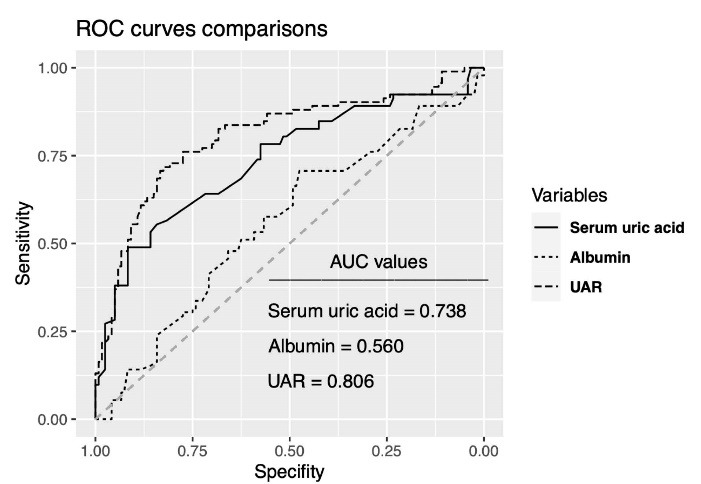
ROC curve comparisons of variables for detecting poor coronary collateral circulation

**Figure 2:**
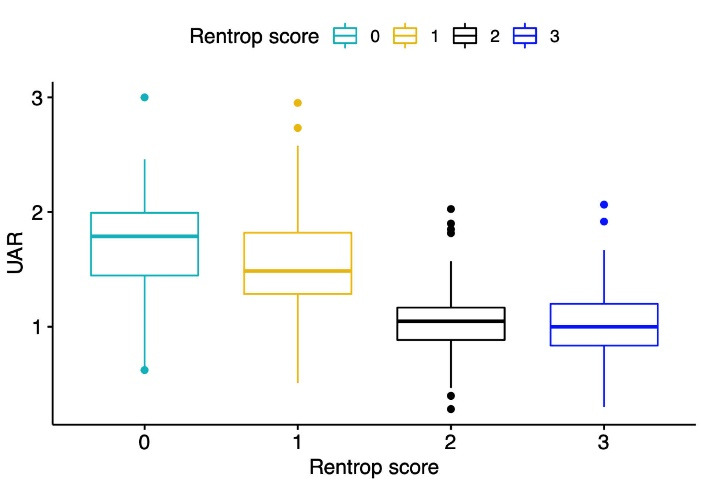
Box-plot graph of uric acid/albumin ratio levels according to rentrop grade scores

 Univariable logistic regression analysis showed that DM, heart failure, lymphocyte, triglyceride, HDL cholesterol, serum UA, UAR, Syntax and Gensini scores, LVEF, LVESD, LVEDD, TDI lateral and septal e waves, and the number of diseased vessels were associated with poor CCC. Multivariable logistic regression analyses revealed that the presence of DM, lymphocyte, triglyceride, UAR, CTO vessel, and LVEF were the variables independently associated with poor CCC (Table 3). Box-plot graphic demonstrated that low Rentrop grades had higher UAR levels than high rentrop grades (Figure 1). An optimal cutoff point of 1.32 for UAR was obtained for the detection of poor CCC using the Youden index (sensitivity = 72%, specificity = 83 %). ROC curve comparison represented that UAR (AUC = 0.806) had a higher discriminative ability than serum UA (AUC = 0.738) and albumin (AUC = 0.560) in detecting poor CCC patients (p values < 0.05 for both comparisons of UAR with serum UA and albumin) (Figure 2).

**Table 3:** Logistic regression analysis of variables for predicting poor CCC

	**Univariable**			**Multivariable**		
	**OR**	**95% CI**	***P*** ** value**	**OR**	**95% CI**	***P*** ** value**
Diabetes mellitus	2.111	1.174- 3.833	0.013	2.813	1.018- 8.123	0.049
Lymphocyte	0.600	0.473- 0.753	< 0.001	0.654	0.428- 0.980	0.043
Triglyceride	1.006	1.003- 1.009	< 0.001	1.010	1.006- 1.015	< 0.001
HDL cholesterol	0.970	0.945- 0.997	0.030	-	-	-
Uric acid	2.676	2.078- 3.605	< 0.001	-	-	-
Albumin	0.783	0.464- 1.313	0.354	-	-	-
UAR	26.920	11.290- 73.350	< 0.001	13.680	4.784- 46.230	< 0.001
Syntax Score	1.105	1.066- 1.151	< 0.001	-	-	-
Gensini Score	1.025	1.011- 1.040	< 0.001	-	-	-
Heart rate	0.980	0.960- 1.003	0.090	-	-	-
LVEF	0.836	0.789- 0.880	< 0.001	0.880	0.816- 0.940	< 0.001
LVEDD	1.062	1.021- 1.107	0.003	-	-	-
TDI lateral e’	0.873	0.784- 0.97	0.010	-	-	-
IVRT	1.016	1.000- 1.034	0.054	-	-	-
Number of diseased vessels	1.473	1.178- 1.864	0.001	-	-	-
CTO vessels						
LAD	Ref	Ref	Ref	Ref	Ref	Ref
Cx	0.967	0.559-1.675	0.922	3.057	1.028- 9.834	0.050
RCA	0.357	0.189-0.665	0.007	0.220	0.052- 0.833	0.031

Abbreviations: CCC, coronary collateral circulation; OR, odds ratio; CI, confidence interval; HDL, high-density lipoprotein; UAR, uric acid/albumin ratio; LVEF, left-ventricular ejection fraction; LVEDD, left-ventricular end-diastolic diameter; TDI, Tissue-Doppler imaging; IVRT, isovolumetric relaxation time; CTO, chronic total occlusion; LAD, left anterior descending; Cx, circumflex; RCA. right coronary artery. *P* < 0.05 statistically significant.

## Discussion

 This study has identified that UAR was higher in patients with poor CCC than in those with good CCC. Furthermore, UAR was an independent predictor of poor CCC in patients with CTO.

 Coronary collaterals occur in long-term chronic coronary artery occlusion and are considered as a compensatory mechanism to feed the ischemic area.^16^ Coronary collaterals improve myocardial contractile activity, decrease infarct size, and promote a positive remodeling phase following a total occlusion of a related coronary artery.^17^ It has been reported in a meta-analysis that the risk of mortality was 36% lower in CAD patients with high collateralization than with low collateralization.^18^ However, although coronary collaterals develop in every patient with CTO, the extent to which they will develop varies from patient to patient. Several studies have focused on this issue and the underlying pathologic difference might be due to altered inflammatory response, oxidative stress, nitric oxide levels, age, the presence of DM, dyslipidemia, and genetic factors.^8,19-22^ It has been reported that the presence of DM was associated with an increased risk of poor CCC.^7,8,23^ In this study, poor CCC patients had a higher frequency of DM, and DM was detected as independently associated with the presence of poor CCC. Kadi et al reported that poor CCC patients had lower HDL than good CCC patients, and HDL was a predictor of poor CCC.^3^ In accordance with this study, HDL was lower in the poor CCC group in our study but HDL did not remain significantly associated with poor CCC in multivariable regression analysis. Liu et al demonstrated that triglyceride was higher in poor CCC patients and triglyceride/HDL ratio was a predictor of poor CCC.^24^ Similarly, our study showed that poor CCC patients had higher triglyceride levels than good CCC patients and triglyceride level was an independent predictor of poor CCC. It was stated that LVEF did not change between poor and good CCC groups in most studies.^7-9^ Nacar et al reported lower LVEF values in patients with poor CCC compared to good CCC patients.^23^ Karpanau et al showed that poor CCC patients had lower LVEF when compared to good CCC patients, which was in concordance with our results. Moreover, our study revealed that LVEF was an independent predictor of poor CCC. Previous studies did not report differences between CCC groups with respect to related CTO vessels.^2,8,9,25^ Our study revealed that poor CCC patients had higher CTO rates in Cx and LAD arteries and lower CTO rates in RCA and the risk of poor CCC development in RCA CTO was lower and in Cx CTO was higher with statistically borderline when compared to LAD CTO. Similar results were reported in previous studies, in which RCA had higher rates and Cx had lower rates of good CCC.^26,27^ The possible explanation of this finding might be due to the size of the coronary artery that supplies collateralization to the total occluded artery. LAD is the biggest coronary artery in the heart and supplies the majority of collaterals to RCA which results in higher collateralization in the presence of the total occlusion of the RCA.^27^ McEntegart et al reported that the lowest collateralization was observed in the Cx total occlusion.^28^ They reported that the main collaterals of the Cx occlusion were firstly supported from the diagonal artery of LAD (32.2 %) and secondly from the posterior left ventricular branch of the Cx artery (20.7 %), whereas the septal branches of LAD were the main arteries that support main collaterals in RCA total occlusion (72%), which might be the possible underlying source of difference of collateralization between Cx and RCA total occlusions. But the main underlying reason for this difference could not have been explained clearly yet.

 Albumin acts as a negative acute phase reactant and the levels of albumin decrease by an enhanced inflammatory status.^29^ The higher inflammation was found to be associated with poor CCC status.^7^ Furthermore, it has been proposed that serum albumin acts as an antioxidant in the human body.^30^ There are controversial results of previous studies regarding the relationship between oxidative stress and CCC development. Demirbag et al found that poor CCC was in relation with low oxidative stress.^31^ But conversely, Gu et al reported that antioxidants stimulate the development of CCC.^32^ Finally, Rocic et al proposed that both too high or too low oxidative stress could decrease coronary collateral growth.^33^ Knoshita et al revealed that serum albumin was negatively correlated with oxidative stress.^34^ Additionally, lower serum albumin levels were associated with cardiovascular diseases and were independent predictors of cardiac adverse events.^35^ Kurtul et al showed that serum albumin was associated with the extent of CAD.^36^ The relationship between serum albumin and CCC has been shown in previous research. The poor CCC patients had lower serum ischemia-modified albumin levels and albumin was an independent predictor of poor CCC.^8^ However, Kelesoglu et al found no difference between poor and good CCC regarding serum albumin levels. In this study, although serum albumin level was lower in the poor CCC group than in the good CCC group, there was no statistically significant difference between the groups.

 Serum UA is a final product of purine metabolism and has a key role in the development and progression of CAD.^2^ Hyperuricemia increases oxidative stress and activates inflammation, which might lead to endothelial dysfunction, microvascular damage, and atherosclerosis resulting in poor CCC development.^2,37^ Uysal et al found that serum UA was higher in poor CCC and was an independent predictor of CCC in stable CAD patients.^38^ Similarly, Kasapkara et al reported that patients with high serum UA levels had higher rates of poor CCC in the non-ST segment elevation (NSTEMI) patients.^37^ In accordance with these studies, serum UA was higher in the poor CCC group and was associated with poor CCC in this study.

 The UAR has emerged as a novel marker that was recently reported as associated with cardiovascular diseases. We have recently reported that UAR was associated with contrast-induced nephropathy in ST-segment elevation myocardial infarction patients (STEMI) following primary percutaneous coronary intervention.^11^ Kalkan et al showed that UAR was an independent predictor of mortality in STEMI patients.^10^ Cakmak et al found that UAR was related to the extent of coronary artery stenosis in NSTEMI patients.^39^ Due to high UA and low serum albumin levels being to be associated with poor CCC, we aimed to investigate whether the ratio of those two variables might better detect poor CCC than the variables alone in this study. The UAR was superior to both serum UA and serum albumin in detecting poor CCC in the current study. Because detecting CTO patients with poor CCC has an important clinical and prognostic implication, the UAR might be used for this purpose better than the serum UA and albumin.

 Our study had some limitations as follows; (1) retrospective study design with a small sample size was the major limitation, (2) due to the cross-sectional study design a causative relationship between UAR and CCC could not be described and more information should be gathered for this purpose in future studies (3) there might be unmeasured confounding variables that might affect the UAR in this study, (4) due to the measurement of variables was obtained upon admission, the impact of following values of variables on CCC could not be reported.

## Conclusion

 This study’s results indicated that the UAR was associated with poor CCC in CTO patients. Of note, the predictive ability of UAR was higher than its components, including serum UA and albumin.

## Acknowledgments

 None.

## Competing Interests

 The authors declared no conflict of interest.

## Ethical Approval

 The local ethic committee approved this study. (Approval number: 2022/15-04).

## Funding

 This research received no specific grant from any funding agency in the public, commercial, or not-for-profit sectors.
